# Reliability and Validity of Single Axial Slice vs. Multiple Slice Quantitative Measurement of the Volume of Effusion-Synovitis on 3T Knee MRI in Knees with Osteoarthritis

**DOI:** 10.3390/jcm12072691

**Published:** 2023-04-04

**Authors:** Greg Gilles, Arjun Vohra, Dagoberto Robles, Mihra S. Taljanovic, Erin L. Ashbeck, Chelsea Caruso, Jeffrey Duryea, Edward J. Bedrick, Ali Guermazi, C. Kent Kwoh

**Affiliations:** 1 University of Arizona Arthritis Center, University of Arizona College of Medicine, Tucson, AZ 85724, USA; greggilles@arizona.edu (G.G.); vohraa@arizona.edu (A.V.); roblesdagoberto@arizona.edu (D.R.); mihrat@radiology.arizona.edu (M.S.T.); eashbeck@arthritis.arizona.edu (E.L.A.); chelseaisme777@gmail.com (C.C.); 2 Departments of Medical Imaging and Orthopaedic Surgery, University of Arizona, Tucson, AZ 85719, USA; 3 Department of Radiology, University of New Mexico Health Sciences, Albuquerque, NM 87131, USA; 4 Tualatin Imaging P.C., Tualatin, OR 97062, USA; 5 Department of Radiology, Brigham and Women’s Hospital, Harvard Medical School, Boston, MA 02115, USA; jduryea@bwh.harvard.edu; 6 Department of Epidemiology and Biostatistics, Mel and Enid Zuckerman College of Public Health, University of Arizona, Tucson, AZ 85724, USA; edwardjbedrick@arizona.edu; 7 VA Boston Healthcare System, West Roxbury, Boston, MA 02132, USA; guermazi@bu.edu; 8 Department of Radiology, Boston University School of Medicine, Boston, MA 02118, USA

**Keywords:** MRI, osteoarthritis, quantitative imaging, knee, biomarkers, effusion, synovitis, inflammation, semi-automated

## Abstract

Effusion-synovitis (ES) is recognized as a component of osteoarthritis, creating a need for rapid methods to assess ES on MRI. We describe the development and reliability of an efficient single-slice semi-automated quantitative approach to measure ES. We used two samples from the Osteoarthritis Initiative (OAI): 50 randomly selected OAI participants with radiographic osteoarthritis (i.e., Kellgren–Lawrence (KL) grade 2 or 3) and a subset from the Foundation for the National Institutes of Health Osteoarthritis Biomarker study. An experienced musculoskeletal radiologist trained four non-expert readers to use custom semi-automated software to measure ES on a single axial slice and then read scans blinded to prior assessments. The estimated intraclass correlation coefficient (ICC) for intra-reader reliability of the single-slice ES method in the KL 2–3 sample was 0.96 (95% CI: 0.93, 0.97), and for inter-reader reliability, the ICC was 0.90 (95% CI: 0.87, 0.95). The intra-reader mean absolute difference (MAD) was 35 mm^3^ (95% CI: 28, 44), and the inter-reader MAD was 61 mm^3^ (95% CI: 48, 76). Our single-slice quantitative knee ES measurement offers a reliable, valid, and efficient surrogate for multi-slice quantitative and semi-quantitative assessment.

## 1. Introduction

Osteoarthritis (OA), particularly knee OA, is a highly prevalent joint disease and a leading source of chronic pain, disability, and economic burden [1,2,3]. With the increasing age, obesity, and sedentary lifestyle of the population, the incidence and burden of knee OA will continue to grow [4,5,6]. No disease-modifying treatments for OA have been approved by the US Food and Drug Administration (FDA) or European Medicines Agency (EMA) [7]. There is a critical need to develop and evaluate biomarkers of knee OA for the purposes of identifying appropriate participants for clinical trial enrollment and ascertaining outcomes during trial follow-up.

The development of osteophytes and the degradation of articular cartilage traditionally characterized the pathogenesis of OA, which is now known to be a disease of the whole joint and potentially involves all tissues in and around the joint [8,9,10]. Inflammation of the synovial membrane (effusion and synovitis) is recognized in the pathogenesis of OA and linked with pain, severity, and the development and progression of knee OA [11,12,13,14]. MRI has evolved as an increasingly important imaging modality in OA research with great clinical and translational potential [15], largely due to its ability to visualize soft tissues. Contrast-enhanced MRI (CE-MRI), such as with the use of gadolinium-based contrast agents (GBCAs), is the gold standard to assess inflammation and is able to differentiate between effusion and synovitis [16]. Due to limitations in added cost, time, complexity, possibility of gadolinium accumulation in various tissues [17,18,19], and contraindications in patients with renal dysfunction and cost–benefit concerns [20], CE-MRI is not often used. The assessment of effusion and synovitis as a combined entity on non-contrast-enhanced (NCE) MRI (i.e., effusion-synovitis (ES)) is a widely used alternative [20,21]. ES has been used broadly as a synovial inflammation-related biomarker on NCE-MRI. ES on MRI has been correlated with radiographic knee OA development [22,23], disease progression [24,25], pain [26], cartilage loss [27,28], and total knee replacement (TKR) [29]. The inflammatory phenotype has been identified as one of six main clinical phenotypes of knee OA [30].

A number of semi-quantitative scores of MRI features (i.e., WORMS, BLOKS, MOAKS, and OMERACT KIMRISS), including ES, have been developed for use in OA research [31]. These ordinal scoring systems require time-consuming and subjective assessment of MRI by expert readers and, therefore, are resource-intensive and costly. Semi-automated and fully-automated quantitative volumetric methods to measure ES have been developed to generate more objective and granular data, but they rely primarily on expert measurements [32,33,34,35]. These methods depend on static thresholding to segment voxels as ES without regard to patient-specific and scan-to-scan variation in presentation, which can ultimately influence appropriate threshold levels required for high precision and reliability. Thus, solutions are needed to leverage the advantages of the participation of readers without formal musculoskeletal radiology training to efficiently produce quantitative measurements of large data sets.

The purpose of this study was to develop a quantitative measurement of ES based on the use of the single axial slice with the largest area of ES. We evaluated the reliability of the new single-slice quantitative measurement of ES and its correlation with previously validated multi-slice quantitative and semi-quantitative methods of ES measurement.

## 2. Materials and Methods

### 2.1. Study Design, Setting, Participants

The Osteoarthritis Initiative (OAI) is a longitudinal cohort study of 4796 individuals, aged 45–79 years, recruited between 2004 and 2006 from the University of Maryland School of Medicine and Johns Hopkins University (Baltimore, MD, USA), Ohio State University (Columbus, OH, USA), University of Pittsburgh (Pittsburgh, PA, USA) and Memorial Hospital of Rhode Island (Pawtucket, RI, USA). The OAI study protocol, images, data, and documentation are available online (https://nda.nih.gov/oai, accessed on 23 November 2022).

For the current reliability study, we used two samples from the OAI: (1) 50 randomly selected OAI participants with radiographic osteoarthritis in at least one knee at baseline, identified as Kellgren–Lawrence (KL) grade 2 or 3 [36], with an existing semi-quantitative assessment of effusion-synovitis on MRI from ancillary studies performed inside the OAI. This sample of 50 participants included one knee per participant, selected with maximum KL grade, or otherwise randomly if both knees had the same KL grade. (2) A subset of 301 knees drawn from the 600 participants in the Foundation for the National Institutes of Health (FNIH) Osteoarthritis Biomarkers Consortium, which was designed as a nested case–control study within the OAI [37]. The subset was chosen randomly for prior MRI measurement methodology development and was designed to follow the case and control distributions of the full FNIH sample [38]. The FNIH subset included radiographic and pain progressors (n = 97), radiographic-only progressors (n = 52), pain-only progressors (n = 52), and non-progressors (n = 100) (we had access to the raw data from this study) [38,39].

Four non-expert readers (G.G., C.C., A.V., D.R.) were trained to use custom semi-automated software to measure ES on a single axial slice under the direction of a musculoskeletal (MSK) radiologist with 30 years of experience in musculoskeletal MRI, and then read scans from the KL 2–3 sample (n = 50), blinded to prior assessments (two replicates for three readers, one replicate for one reader). The amount of time required to train a non-expert reader ranged from ~2 to ~4 h with a training set of 50 scans. One non-expert reader also measured ES on a single axial slice in the FNIH subset (n = 301) to enable comparison with the multi-slice methodology [38].

### 2.2. Radiography Acquisition and Kellgren–Lawrence Grade Assessment

Bilateral posteroanterior fixed-flexion weight-bearing radiographic views were obtained using a SynaFlexer (Synarc, San Francisco, CA, USA), as described in the radiographic procedure manual (https://nda.nih.gov/oai, accessed on 23 November 2022). Expert readers centrally scored the images using the KL grading system [36], with adjudication by an MSK radiologist (more details can be found from the OAI documentation [40]).

### 2.3. MRI Acquisition and Semi-Quantitative Scoring Assessment of Effusion-Synovitis

Non-contrast-enhanced MRI scans were acquired from the 4 OAI sites on identical 3 Tesla (T) systems (Siemens Trio MR, Erlanger, Germany). MSK radiologists centrally reviewed the scans and graded ES using the MRI Osteoarthritis Knee Score (MOAKS) (more details can be found from the OAI documentation [41]). The MOAKS ES grade is a whole-scan, semi-quantitative assessment based on hyperintensity within the articular cavity that represents a composite of effusion and synovial thickening (0: physiologic amount; 1: small—fluid continuous in the retropatellar space; 2: medium—with slight convexity of the suprapatellar bursa; 3: large—evidence of capsular distention). The reported intra-rater and inter-rater reliability of MOAKS ES, scored by MSK radiologists, was 0.90 (95% CI: 0.78, 1.00) and 0.72 (95% CI: 0.52, 0.92), respectively, calculated using linear weighted kappa [42].

### 2.4. Semi-Automated Quantitative Assessment of Effusion-Synovitis

**Figure 1:**
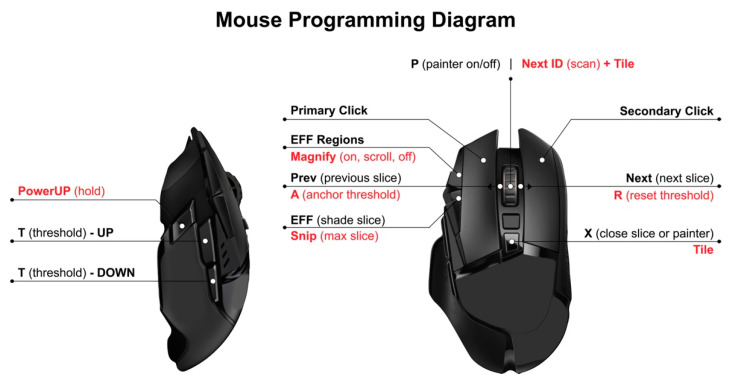
Diagram of mouse displaying 20 software functions programmed as buttons that can be operated rapidly to generate precise effusion-synovitis shadings and subregional divisions.

The sagittal 3D dual-echo steady-state sequence (DESS) with water excitation was reformatted to axial 3T DESS images that were assessed for ES. A customizable software platform that incorporated and revised aspects of a previous semi-automated approach was developed [38]. The team used commercially available hardware and software tools (i.e., i7 desktop, DICOM-rated 27″ 4K monitor, and gaming mice) to build an optimized workspace that could be rapidly deployed for use at remote sites. A single-slice approach was chosen to reduce the time it took to assess each knee for ES. The process was further enhanced by programming a multi-button mouse to perform all 20 required functions that were part of the semi-automated method to allow the use of one hand to perform all of the functions without needing to look away from the area of focus (see Figure 1). Our team decided to confine the choice of a single axial 3T DESS slice within the region located between the superior and inferior poles of the patella, as previous literature has demonstrated that ES knee OA is primarily present within this region [43]. Additionally, this allowed the methodology to maintain consistency with a prior reported semi-automated multi-slice method for segmenting ES [38]. The final system employed several desktop automations that included lossless magnifiers (toggling/adjusting full-picture zoom; continuous focal magnification) and local image capturing to compare original images with saved mappings while revising as needed.

**Figure 2:**
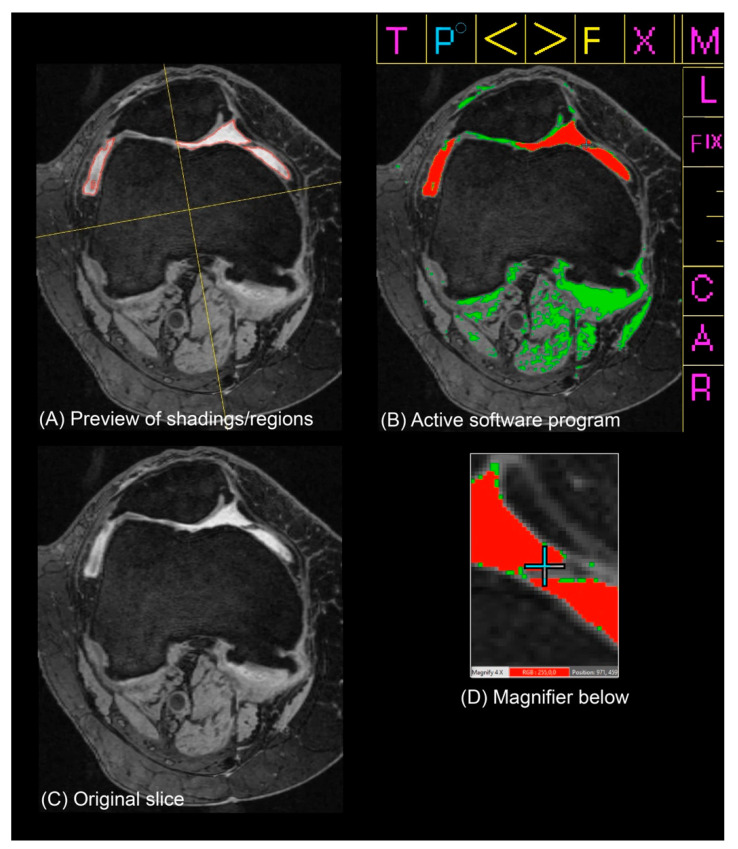
Software system and effusion-synovitis (ES) measurement method used to generate ES shadings and subregional divisions and verify/revise areas of fine detail. Panels (**A**–**D**) were viewed simultaneously by a reader in a single display (without the captions). (**A**) Preview of shadings and regions generated in the top left portion of the screen. (**B**) Active software program in the top right corner of screen. (**C**) Original slice below the preview of shadings/regions. (**D**) Magnifier on the cursor below the active program.

The measurement procedure began with displaying all 60 axial slices in a 10 × 6 tile on a single 27″ 178° wide-viewing angle screen. After loading all of the axial slices, the superior and inferior poles of the patella were identified and marked on their respective slices. Within the range of the patellar groove, the slice judged to contain the largest area of ES was selected. Leveraging the region-growing algorithms and dynamic, variable grayscale thresholding of the program, the reader would typically settle on a threshold encompassing potential pixels that were consistent with the presence of ES. After selecting the more prominent regions of effusion, the reader would sequentially toggle the sensitivity and adjust finer details. Fluid was captured in the patellofemoral joint (PFJ), along the trochlea and extending into the lateral and medial recesses and posteriorly around the femoral condyles. Lastly, subregional divisions that were anchored to the femur using the MOAKS delineation (anterior vs. posterior; medial vs. lateral) were drawn to segment the ES into one of four quadrants (AM, AL, PM, PL) [42]. After confirming a final review of the segmented slice enlarged, the reader would proceed to the next scan (see Figure 2).

### 2.5. Statistical Analysis

Participant and knee-level characteristics from the OAI baseline visit were summarized for the KL 2–3 sample (n = 50) and the FNIH subset (n = 301). Distributions of single-slice measurements of ES were plotted and summarized in each sample separately.

Reliability was evaluated based on the intraclass correlation coefficient (ICC), defined as the proportion of the total variance in the measurements due to “true” differences between subjects, where the “true” value is the average that would be obtained if measured an infinite number of times. This value reflects the consistency of the measurement, not the accuracy. While the ICC reflects how well subjects can be distinguished from each other despite the presence of measurement error, it is a relative measure that depends on the heterogeneity of the sample; subjects in a heterogeneous population are easier to distinguish than subjects who are similar in terms of the feature being measured. The standard error of measurement (SEM) is an absolute measure of how far apart repeated measurements are for a single subject, expressed in the unit of measurement [44,45]. Intra- and inter-reader ICC, as well as the SEM, were estimated from a linear mixed model, with random effects for knee, reader, and the interaction between knee and reader (see Supplementary Materials for model and the ICC and SEM formulas). Bias-corrected and acceleration-adjusted (BCa) nonparametric bootstrap 95% confidence intervals (95% CI) were generated from 20,000 replicates, with resampling at the knee level [46,47].

We estimated the intra-reader mean absolute difference (MAD), defined as the mean difference between two measurements by the same reader, and the inter-reader MAD, defined as the mean difference between any two measurements by different readers. The 95% CIs were generated from bootstrap nonparametric percentiles from 20,000 replicates [48,49].

Concurrent criterion validity of the single-slice method was evaluated based on comparison to the multi-slice method, as well as ES grading by MSK radiologists. We estimated the Spearman correlation between total ES measured on a single axial slice and total ES volume measured with the multi-slice methodology [38] in the FNIH subset (n = 301), as well as the correlation between the single-slice ES measurement and MOAKS ES in the FNIH subset (n = 301) and the KL 2–3 sample (n = 50). BCa bootstrap 95% CIs were generated from 20,000 replicates [50].

We compared the contributions of MOAKS ES, quantitative multi-slice ES measurement, and quantitative single-slice ES measurement by comparing their contributions to FNIH case status [37]. We fit a logistic regression model for radiographic and pain progression case status (97 cases vs. 204 controls) with the following predictors: KL grade, BMI, sex and age, MOAKS ES, multi-slice ES, and single-slice ES (Model XABC). To test the null hypothesis that MOAKS ES provides no additional information beyond the two quantitative methods (multi-slice and single-slice), we compared the full model to a reduced model that did not include the MOAKS ES variable (Model XBC) with a likelihood ratio test. To test the null hypothesis that quantitative ES measurement (multi-slice and single-slice) provides no additional information beyond MOAKS ES, we compared the full model to a reduced model that did not include the quantitative ES variables (Model XA). Finally, to compare information provided by the two quantitative ES methods, we compared a model that included multi-slice and single-slice ES measurements (Model XBC) to a reduced model that did not include the multi-slice ES predictor (Model XC) and a reduced model that did not include single-slice ES (Model XB). Using the same approach, we compared the contributions of MOAKS ES and the two quantitative ES methods to radiographic progression case status (149 cases vs. 152 controls), and for pain progression case status (149 cases vs. 152 controls).

## 3. Results

The KL 2–3 (n = 50) sample included participants identified as Non-Hispanic White (88%) and African American (12%), with 66% reporting female sex. The mean participant age was 62 years (SD 9), and mean BMI was 30 kg/m^2^ (SD 5). The knees were graded as follows: KL 2 (62%) and KL 3 (38%), medial Osteoarthritis Research Society International (OARSI) joint space narrowing (JSN) grade 1 (20%) and 2 (30%), and lateral OARSI JSN 1 (2%) and 2 (8%). Frequent knee pain was reported for 41% of the knees, with a median Western Ontario and McMaster Universities OA Index (WOMAC) knee pain score of 2 (Q1, Q3: 0, 4). Knees in the FNIH subset (n = 301) were graded KL 1 (15%), KL 2 (54%), and KL 3 (31%), though with greater prevalence of medial JSN grade 1 (37%) and 2 (31%), and less prevalence of lateral JSN grade 1 (2%). In the FNIH subset, 30% reported frequent knee pain, with a median WOMAC knee pain score of 1 (Q1, Q3: 0, 3). Distributions of participant-level demographics in the FNIH subset were roughly similar to those in the KL 2–3 sample (Table 1).

The estimated ICC for intra-reader reliability of the single-slice ES method in the KL 2–3 sample was 0.96 (95% CI: 0.93, 0.97), and for inter-reader reliability, the ICC was 0.90 (95% CI: 0.87, 0.95). The corresponding estimates for measurement error were SEM 41.5 mm^3^ (95% CI: 39.3, 52.5) within reader and SEM 68.3 mm^3^ (95% CI: 50.3, 81.1) when accounting for differences between readers. The intra-reader MAD was 35 mm^3^ (95% CI: 28, 44), and the inter-reader MAD was 61 mm^3^ (95% CI: 48, 76) (Table 2).

**Figure 3:**
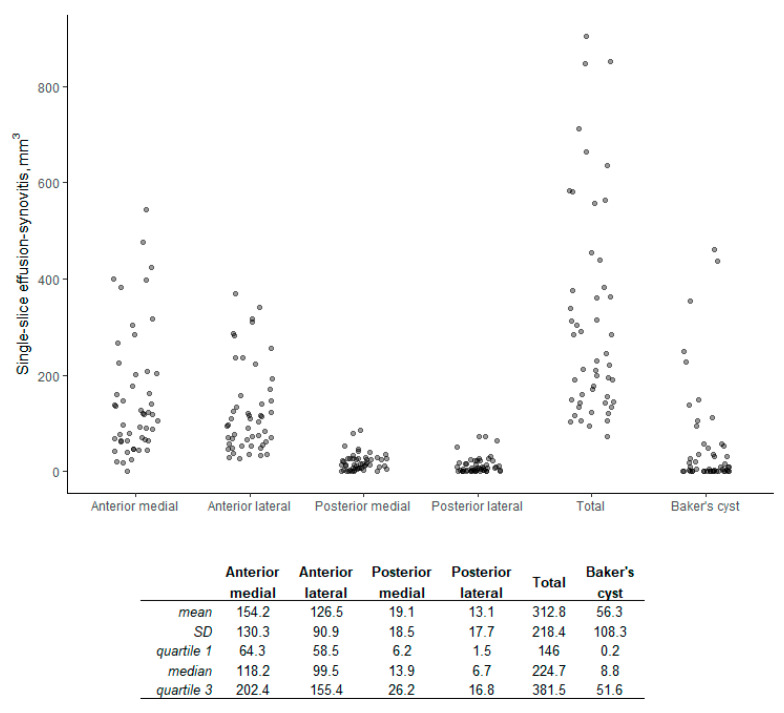
Distribution of single-slice volume with summary statistics (KL 2–3 sample, n = 50).

After a ramp-up period of segmenting approximately 50 scans, average reading time was calculated as less than 2 min per scan by the reader most familiar with the tools. Subregional and total single-slice ES measurements in the KL 2–3 sample, as well as single-slice measurement of Baker’s cysts, averaged across the readers, are shown in Figure 3. Single-slice ES measurements in the FNIH subset are shown by case–control status in Figure S1.

**Figure 4:**
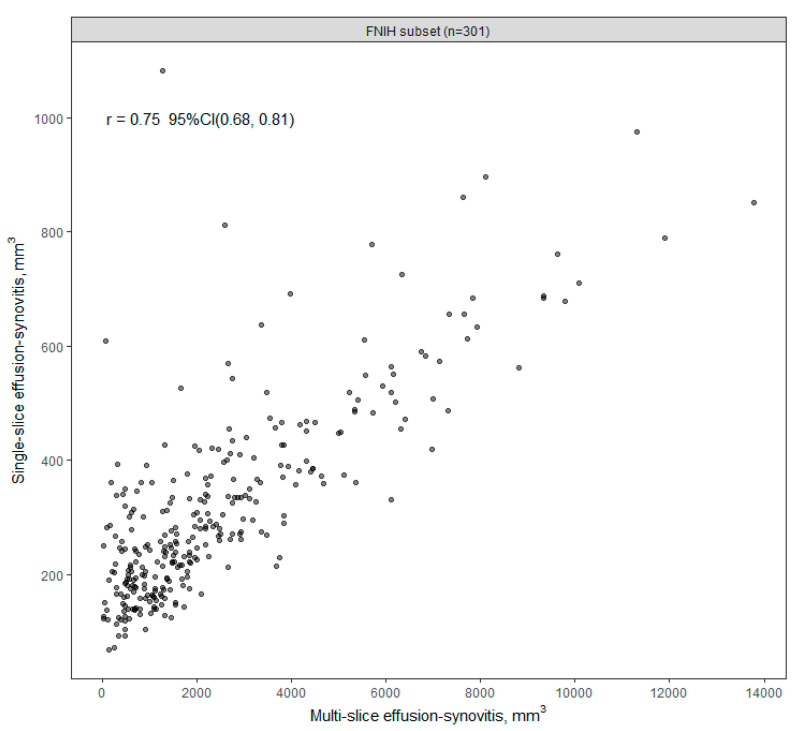
Single-slice vs. multi-slice effusion-synovitis volume (FNIH, n = 301).

**Figure 5:**
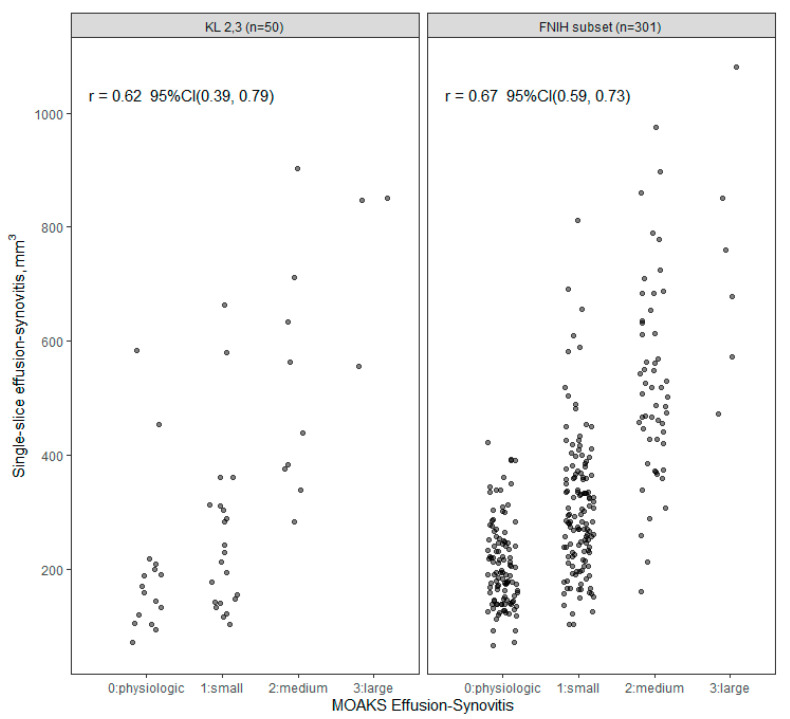
Single-slice effusion-synovitis (ES) volume vs. MOAKS (MRI Osteoarthritis Knee Score) ES.

A comparison between total ES measured on a single axial slice and total ES volume measured with the multi-slice methodology in the FNIH subset is shown in Figure 4, with an estimated correlation of 0.75 (95% CI: 0.68, 0.81). The single-slice ES measurements are compared with MOAKS ES in Figure 5, with an estimated correlation of 0.62 (95% CI: 0.39, 0.79) in the KL 2–3 sample, and 0.67 (95% CI: 0.59, 0.73) in the FNIH subset. The multi-slice ES volume measurement was compared to MOAKS ES in the FNIH subset previously, with an estimated correlation of 0.74 (95% CI: 0.68, 0.79) (Figure S2).

In the FNIH case–control subset, we found that both quantitative ES methods, multi-slice and single-slice ES measurement, provided information beyond MOAKS ES for radiographic and pain progression case status (LR 8.6, *p* = 0.01). MOAKS ES did not significantly improve the model fit for radiographic and pain progression case status beyond the quantitative ES methods (LR 2.3, *p* = 0.51). The single-slice ES measurement provided information beyond the multi-slice measurement (LR 6.9, *p* < 0.01), while the multi-slice ES measurement was not significant in a model that already included the single-slice ES measurement (LR 2.3, *p* = 0.13). For radiographic progression case status, we similarly found that the quantitative ES methods provided additional information beyond MOAKS ES (LR 10.6, *p* < 0.01), with the single-slice ES measurement providing more information than the multi-slice measurement (LR 7.4, *p* < 0.01). When considering pain progression case status, both the multi-slice and single-slice measurements provided added information (LR 10.0, *p* < 0.01 and LR 9.90, *p* < 0.01) (Table 3).

## 4. Discussion

We generated rapid, reproducible ES calculations with strong intra- and inter-reader reliabilities. The single-slice ES measurement had high correlation with the quantitative multi-slice method and with semi-quantitative MOAKS assessed by MSK radiologists in the KL 2–3 and FNIH samples, supporting the concurrent criterion validity.

The reliability of different methods of assessing ES on NCE-MRI has been reported for semi-quantitative and quantitative approaches. The reliability of MOAKS ES readings has been reported as weight kappa and percent agreement, 0.90 (95% CI: 0.78, 1.00) and 0.90, respectively, for intra-rater reliability and 0.72 (95% CI: 0.52, 0.92) and 0.70, respectively, for inter-rater reliability [42]. Our intra-rater and inter-rater reliability were excellent compared to those reported from the results of random-effects pooling of intra-reader ICCs and inter-reader ICCs from a systematic review and those reported by Maksymowych for KIMRISS and MOAKS status scores [51,52]. Our ICCs were also similar to those reported by Wang et al. for their method of quantitative measurement of ES [53]. Li et al. reported that their semi-automated method of measuring ES had similarly moderate correlation (r = 0.77) with a semi-quantitative method of assessing ES (i.e., WORMS) [34]. Our single-slice ES segmentation methodology had excellent intra-rater and inter-rater reliability across four non-expert readers and was as good as or better than the published reliability results for semi-quantitative and quantitative methods that have been developed to assess ES.

By utilizing non-expert readers, our method draws upon a larger pool of potential readers. Its direct costs were low despite its technological and practical advantages. Moreover, our approach proposes technical advancements to previous methods of assessing ES that are constrained to a single threshold and are generally more cumbersome to navigate [38]. The most experienced non-expert reader in this project commented that a single threshold was not sufficient for any slice and, thus, was unlikely to suffice for all of the slices in a knee scan. This sentiment, shared by other readers, indicated that the signal emanating from individual pixels might not definitively provide information about its content absent contextual information (i.e., the surrounding pixels’ intensities and composition of tissues). Two illustrative cases (see Figure S3 in the Supplementary Materials) demonstrate the benefit of not using a set threshold when segmenting NCE-MRIs for ES.

Our single-slice method of assessing ES on MRI was rapid and efficient. Given the lack of expertise in reading MRIs for ES and using these novel software tools, there was an associated learning curve. Consequently, reading time decreased over time, which was calculated as less than two minutes per scan after a brief period of training and practice. This represented a major efficiency improvement when compared to our previous experience of approximately 10 min per scan for a multi-slice approach [38] or 25 min per scan if the reader were able to adjust the threshold in the multi-slice approach while measuring ES across the entire region of interest. The relatively extended amount of time for the multi-slice method was due to the need to selectively and sequentially apply different thresholds to appropriately shade regions of ES across each slice. This issue helped motivate the decision to choose a single slice with the largest area of ES in the PFJ. The move to the single-slice method was also facilitated by the ability to display all 60 uncompressed slices in a 10 × 6 grid on a single 178° wide-viewing angle 4K monitor screen. Efficiency was further improved via a suite of additional software functions, automations, and associated programmable mouse button mappings. This allowed the user to rapidly select the maximum ES slice, dynamically increase and decrease the threshold and shade ES with pixel-by-pixel control, draw subregional divisions, review and revise as necessary and advance between scans. Our approach provided the readers with enough control and flexibility to ensure reliable and valid assessment. Our results suggest that it may be possible to assess effusion volume with fewer images. This potential time-saving approach requires further study [38]. Future considerations include the investigation of the performance characteristics of performing MOAKS readings on a single slice, which would also lead to significant time savings.

We utilized the FNIH case–control study to compare the semi-automated single-slice and multi-slice measurement of ES, as well as the MOAKS semi-quantitative measure of ES. The two quantitative ES methods, multi-slice and single-slice ES measurement, contributed information beyond MOAKS ES for radiographic and/or pain progression case status. Further, the single-slice measurement was more informative than the multi-slice measurement when considering radiographic progression case status, though not necessarily for pain progression case status. The relationships between inflammation and radiographic and/or pain progression are complex. It may be that certain areas with inflammation, such as within the suprapatellar bursa, that are not captured as well by our single-slice or the multi-slice methods do not contribute as much relatively to the pathogenesis of pain or radiographic progression. The MOAKS semi-quantitative measure of ES takes ES in all articular subregions into account. It yields only one semiquantitative score for the whole knee and results in a potential diminution of the contribution of ES that is assessed only between the poles of the patella. Roemer et al. found that 11 articular subregions consistently exhibited definite synovitis in a population with mixed radiographic OA severity, and the suprapatellar bursa was the second commonest synovitis site (59.5% of knees) [14]. Alternatively, since on NCE-MRI synovitis cannot be distinguished from effusion, the relative contributions of synovitis and effusion in the infrapatellar region as segmented by the single and the multi-slice methods may be different when compared to synovitis and effusion in other subregions of the knee that are encompassed by the MOAKS methodology. Calculation of MOAKS ES scores for individual regions within the knee may be needed to further understand the comparisons between semiquantitative MOAKS scoring and the quantitative single slice method. Further work is needed to better understand the contribution of ES in different parts of the knee to the pain and/or radiographic progression of knee OA.

This study had several limitations. ES is distributed in a heterogenous fashion within the knee, resulting in different subregional distributions of ES, whereas our proposed methodology focuses on segmenting a single slice, which has the potential to overestimate or underestimate the amount of ES in the whole knee. Moreover, there was variation in the axial MRI slice that readers selected with the largest area of ES. The slice selected differed by an average standard deviation of 1.89 slices among the four different readers. Furthermore, our method was semi-automated and performed by non-expert readers, which resulted in a learning curve in terms of efficiency and reader competence. There are various causes of effusion that may not be easily distinguished from synovitis due to OA on NCE-MRI. Our results may not be generalizable to images that are from different vendors, machines, sequences, etc. Further work is needed to determine whether the responsiveness of the single-slice method is generalizable to assessing change in ES in longitudinal studies.

## 5. Conclusions

In conclusion, our more efficient single-slice quantitative measurement of ES using non-expert readers had excellent intra-and inter-reader reliability and good correlation with the quantitative multi-slice measurements and semi-quantitative ES graded by MSK radiologists. The proposed quantitative single-slice method is an inexpensive and valid assessment of ES that can be used to segment large sets of MRI images.

## Figures and Tables

**Table 1:** Baseline participant and knee characteristics.

Variable	KL 2–3 (n = 50)	FNIH Subset (n = 301)
Age, years [mean, (SD)]	61.6 (9.2)	62.0 (9.2)
Female sex	33 (66%)	189 (63%)
Race, ethnicity		
White NH	44 (88%)	233 (78%)
Black NH	6 (12%)	56 (19%)
Other	0 (0%)	11 (4%)
BMI, kg/m^2^ [mean, (SD)]	29.6 (4.9)	30.2 (4.6)
BMI categories		
Normal	9 (18%)	40 (13%)
Overweight	16 (32%)	116 (39%)
Obese	25 (50%)	144 (48%)
Kellgren–Lawrence grade		
1	0 (0%)	44 (15%)
2	31 (62%)	163 (54%)
3	19 (38%)	94 (31%)
Joint space narrowing, medial		
0	25 (50%)	97 (32%)
1	10 (20%)	110 (37%)
2	15 (30%)	94 (31%)
Joint space narrowing, lateral		
0	45 (90%)	296 (98%)
1	1 (2%)	5 (2%)
2	4 (8%)	0 (0%)
WOMAC pain	2.8 (3.4)	2.2 (3.0)
Frequent knee pain	20 (41%)	90 (30%)

BMI: body mass index, KL: Kellgren–Lawrence grade, FNIH: Foundation for the National Institutes of Health Osteoarthritis Biomarkers Consortium, WOMAC: Western Ontario and McMaster Universities OA Index.

**Table 2:** Measurement differences within and between readers.

	Mean Absolute Difference, mm^3^			
Variable	Intra-Reader	(95% CI)	Inter-Reader	(95% CI)
Total volume, mm^3^	36	(28, 44)	61	(48, 75)
Anterior medial	21	(16, 28)	32	(26, 39)
Anterior lateral	29	(23, 37)	37	(30, 44)
Posterior medial	12	(9, 16)	17	(12, 22)
Posterior lateral	10	(7, 13)	13	(9, 18)
Baker’s cyst	18	(10, 27)	26	(14, 39)

Note: KL 2–3 sample, n = 50 knees.

**Table 3:** Contributions of MOAKS ES, Multi-slice ES volume and Single-slice ES volume to FNIH case–control status.

Case Type	Model/Hypothesis	Likelihood Ratio Χ^2^	d.f.	Adequacy Index *	*p*
**Radiographic and Pain Progression case**					
	X: KL grade + BMI + sex + age	10.2	5	0.51	
	XA: X + MOAKS ES	11.5	8	0.57	
	XB: X + Multi-slice ES	11.0	6	0.54	
	XC: X + Single-slice ES	15.5	6	0.77	
	XAB: X + MOAKS ES + Multi-slice ES	12.0	9	0.60	
	XAC: X + MOAKS ES + Single-slice ES	19.3	9	0.95	
	XBC: X + Multi-slice ES + Single-slice ES	17.9	7	0.89	
	XABC: X + MOAKS ES + Multi-slice ES + Single-slice ES	20.2	10	1.00	
	**Hypothesis (model comparison)**				
	H_o_: No additional information provided by MOAKS ES beyond quantitative methods (Model XABC vs. XBC)	2.3	3		0.51
	H_o_: No additional information provided by quantitative methods beyond MOAKS ES (Model XABC vs. XA)	8.6	2		0.01
	H_o_: No additional information provided by multi-slice ES volume beyond single-slice ES volume (Model XBC vs. XC)	2.3	1		0.13
	H_o_: No additional information provided by single-slice ES volume beyond multi-slice ES volume (Model XBC vs. XB)	6.9	1		<0.01
**Radiographic Progression case**					
	X: KL grade + BMI + sex + age	25.5	5	0.60	
	XA: X + MOAKS ES	31.5	8	0.75	
	XB: X + Multi-slice ES	32.8	6	0.78	
	XC: X + Single-slice ES	40.0	6	0.95	
	XAB: X + MOAKS ES + Multi-slice ES	34.1	9	0.81	
	XAC: X + MOAKS ES + Single-slice ES	42.1	9	1.00	
	XBC: X + Multi-slice ES + Single-slice ES	40.2	7	0.95	
	XABC: X + MOAKS ES + Multi-slice ES + Single-slice ES	42.1	10	1.00	
	**Hypothesis (model comparison)**				
	H_o_: No additional information provided by MOAKS ES beyond quantitative methods (Model XABC vs. XBC)	1.9	3		0.59
	H_o_: No additional information provided by quantitative methods beyond MOAKS ES (Model XABC vs. XA)	10.6	2		<0.01
	H_o_: No additional information provided by multi-slice ES volume beyond single-slice ES volume (Model XBC vs. XC)	0.2	1		0.68
	H_o_: No additional information provided by single-slice ES volume beyond multi-slice ES volume (Model XBC vs. XB)	7.4	1		<0.01
**Pain Progression case**					
	X: KL grade + BMI + sex + age	7.7	5	0.37	
	XA: X + MOAKS ES	9.4	8	0.44	
	XB: X + Multi-slice ES	8.9	6	0.42	
	XC: X + Single-slice ES	8.8	6	0.42	
	XAB: X + MOAKS ES + Multi-slice ES	9.6	9	0.46	
	XAC: X + MOAKS ES + Single-slice ES	15.7	9	0.75	
	XBC: X + Multi-slice ES + Single-slice ES	18.8	7	0.89	
	XABC: X + MOAKS ES + Multi-slice ES + Single-slice ES	21.1	10	1.00	
	**Hypothesis (model comparison)**				
	H_o_: No additional information provided by MOAKS ES beyond quantitative methods (Model XABC vs. XBC)	2.3	3		0.52
	H_o_: No additional information provided by quantitative methods beyond MOAKS ES (Model XABC vs. XA)	11.7	2		<0.01
	H_o_: No additional information provided by multi-slice ES volume beyond single-slice ES volume (Model XBC vs. XC)	10.0	1		<0.01
	H_o_: No additional information provided by single-slice ES volume beyond multi-slice ES volume (Model XBC vs. XB)	9.9	1		<0.01

All models include the following covariates: baseline KL grade, BMI, sex, and age, indicated as ‘X’. * Adequacy index is defined as LR_s_/LR_f_, where LR_f_ is the −2 log likelihood ratio statistic for the full set of predictors in model XABC, and LR_s_ is the −2 log likelihood ratio statistic for the subset of predictors. MOAKS: MRI Osteoarthritis Knee Score.

## Data Availability

Data used in the preparation of this article were obtained from the Osteoarthritis Initiative (OAI) database, which is available for public access at https://nda.nih.gov/oai/ (accessed on 23 November 2022). Additional data analyzed during the current study is available from the corresponding author upon reasonable request.

## Supplementary Materials

The following supporting information can be downloaded at: https://www.mdpi.com/article/10.3390/jcm12072691/s1, Equation S1: Linear mixed model with random effects for knee, reader, and the interaction between knee and reader, Formula S1: Intra-reader intraclass correlation coefficient (ICC), Formula S2: Intra-reader standard error of measurement (SEM), Formula S3: Inter-reader intraclass correlation coefficient (ICC), Formula S4: Inter-reader standard error of measurement (SEM), Figure S1: Single-slice ES volume in FNIH sample: Distribution summary (n = 301), Figure S2: Multi-slice effusion-synovitis volume vs. MOAKS effusion-synovitis (FNIH, n = 301), Figure S3: Two illustrative examples of variable threshold segmentation performance by non-expert readers.
