# Supplementary material for: Reliability and Validity of Single Axial Slice vs. Multiple Slice Quantitative Measurement of the Volume of Effusion-Synovitis on 3T Knee MRI in Knees with Osteoarthritis
Source: J Clin Med. 2023 Apr 4;12(7):2691. doi: 10.3390/jcm12072691 (PMC10095125; doi:10.3390/jcm12072691)
Supplement: Supplementary file 1 [file jcm-12-02691-s001.zip › jcm-2172912-Supplementary Materials.pdf]

Supplementary Materials

Table of Contents

1) Linear mixed model with random effects
2) Intra-reader reliability
3) Inter-reader reliability
4) Distribution of single-slice volume by FNIH case status
5) Multi-slice effusion-synovitis volume vs. MOAKS effusion-synovitis
6) Two cases illustrating the importance of precise variable thresholding

1) Linear mixed model with random effects

Equation S1. Linear mixed model with random effects for knee, reader, and the interaction between knee and reader

$$Y_{ijk} = \mu + knee_i + reader_j + (knee{:}reader)_{ij} + \varepsilon_{ijk},$$

where Y_{ijk} is the kth effusion-synovitis measurement of knee i measured by reader j, $knee_i$ is the random effect of knee I, $reader_j$ is the random effect of reader j, $(knee{:}reader)_{ij}$ is the random interaction between knee i and reader j, ε_{ijk} is the residual.

$$knee_i \text{ i.i.d.} \sim N(0, \sigma_{knee}^2)$$
$$reader_j \text{ i.i.d.} \sim N(0, \sigma_{reader}^2)$$
$$(knee{:}reader)_{ij} \text{ i.i.d.} \sim N(0, \sigma_{knee:reader}^2)$$
$$\varepsilon_{ijk} \text{ i.i.d.} \sim N(0, \sigma_{\varepsilon}^2)$$

2) Intra-reader reliability

Intra-reader reliability was defined as:

1) $ICC_{intra} = [\sigma^2_{knee} + \sigma^2_{reader} + \sigma^2_{knee:reader}] / [\sigma^2_{knee} + \sigma^2_{reader} + \sigma^2_{knee:reader} + \sigma_{\varepsilon}^2]$

Formula S1. Intra-reader intraclass correlation coefficient (ICC)

2) $SEM_{intra} = \sqrt{(\sigma_{\varepsilon}^2)}$

Formula S2. Intra-reader standard error of measurement (SEM)

3) Inter-reader reliability

Inter-reader reliability (within-knee and between-reader) was defined as:

1) $ICC_{inter} = \sigma^2_{knee} \,/\, [\sigma^2_{knee} + \sigma^2_{reader} + \sigma^2_{knee:reader} + \sigma_\varepsilon^2]$

Formula S3. Inter-reader intraclass correlation coefficient (ICC)

2) $SEM_{inter} = \sqrt{(\sigma^2_{reader} + \sigma^2_{knee:reader} + \sigma_\varepsilon^2)}$

Formula S4. Inter-reader standard error of measurement (SEM)

4) Distribution of single-slice volume by FNIH case status

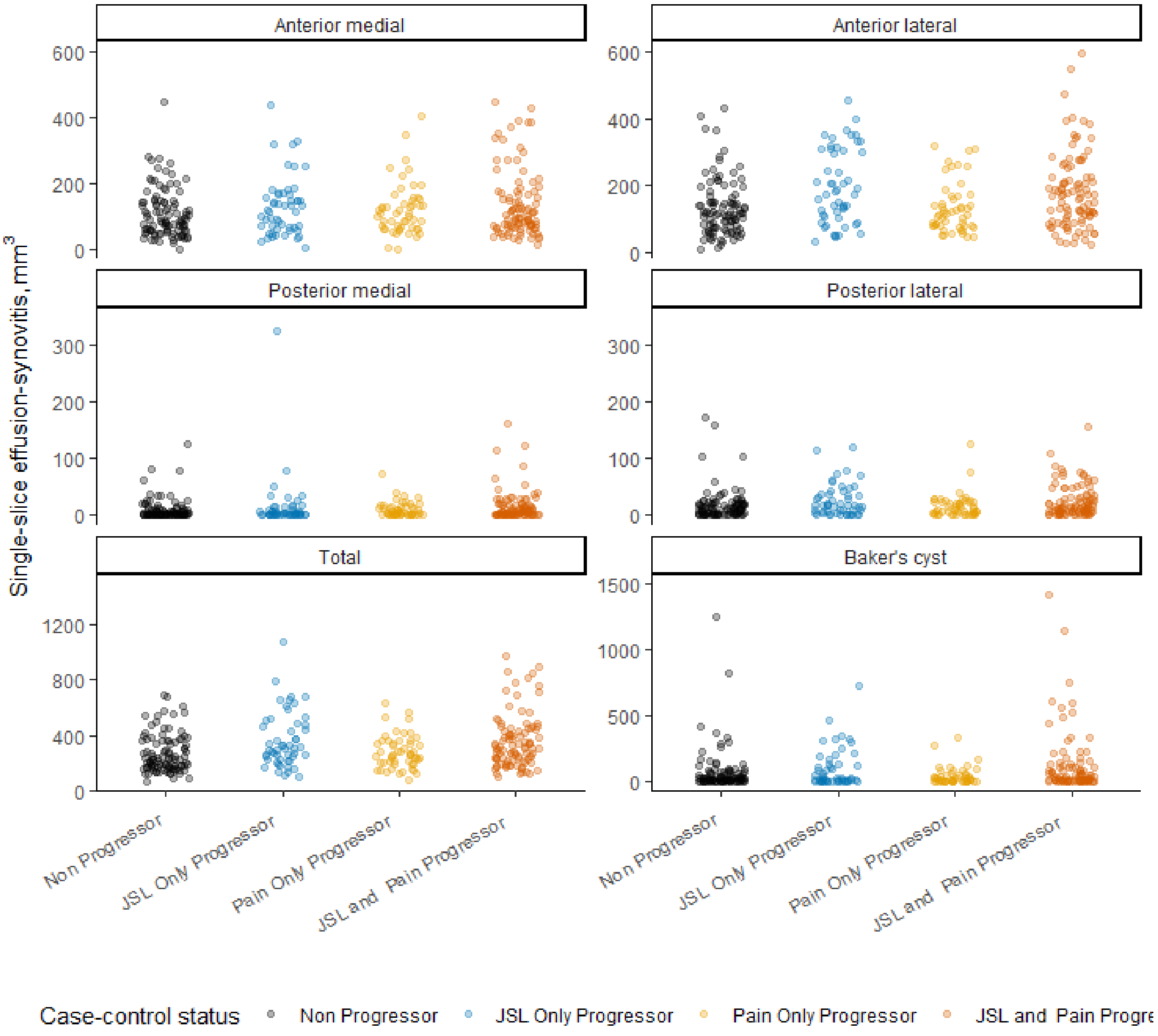

Figure S1. Single-slice effusion-synovitis volume in FNIH sample: Distribution summary (n = 301). JSL: Joint space loss. FNIH: Foundation for the National Institutes of Health Osteoarthritis Biomarkers Consortium.

5) Multi-slice effusion-synovitis volume vs. MOAKS effusion-synovitis

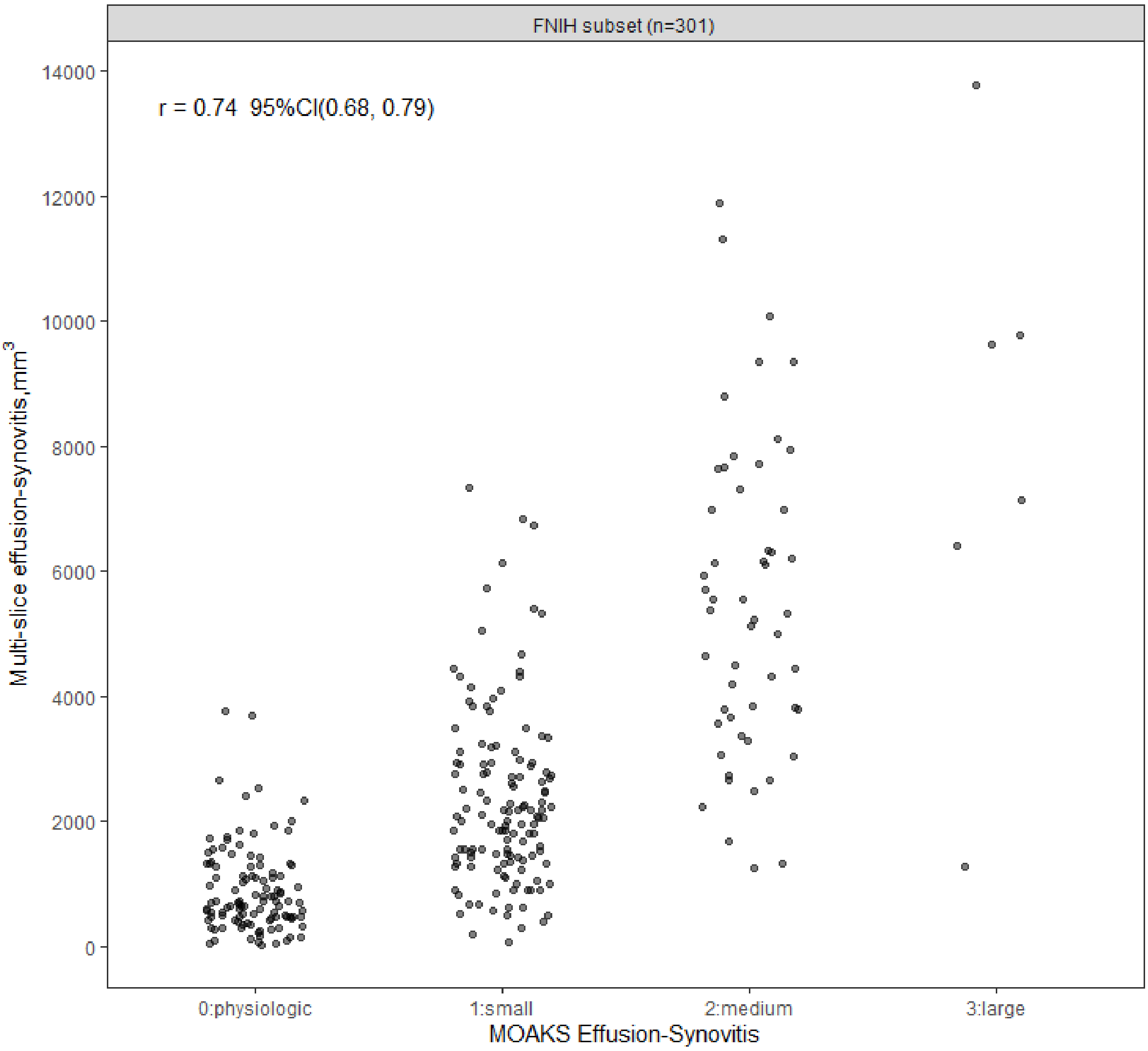

Figure S2. Multi-slice effusion-synovitis volume vs. MOAKS effusion-synovitis (FNIH, n = 301). MOAKS: MRI Osteoarthritis Knee Score.

6) Two cases illustrating the importance of precise variable thresholding

There are two cases that demonstrate the significant benefit of being able to precisely apply multiple thresholds to segment effusion-synovitis (ES) on non-contrast-enhanced MRI.

Case 1: Continuous region of joint fluid displays different signal intensities due to imaging artifact;

Case 2: Similar signal intensity of trochlear cartilage and surrounding ES located medially or laterally;

Problem: Without control of and proper tuning of the threshold, a semi- or fully-automated reading may segment cartilage as ES, or conversely apply too low of a threshold globally and under-capture additional ES regions.

Supplemental Figure 3 below illustrates both cases, as well as how our non-expert readers exhibited improvement in their ability to apply the appropriate dynamic thresholds as they gained more experience. With sufficient experience, our readers displayed high competence in handling these more specialized situations.

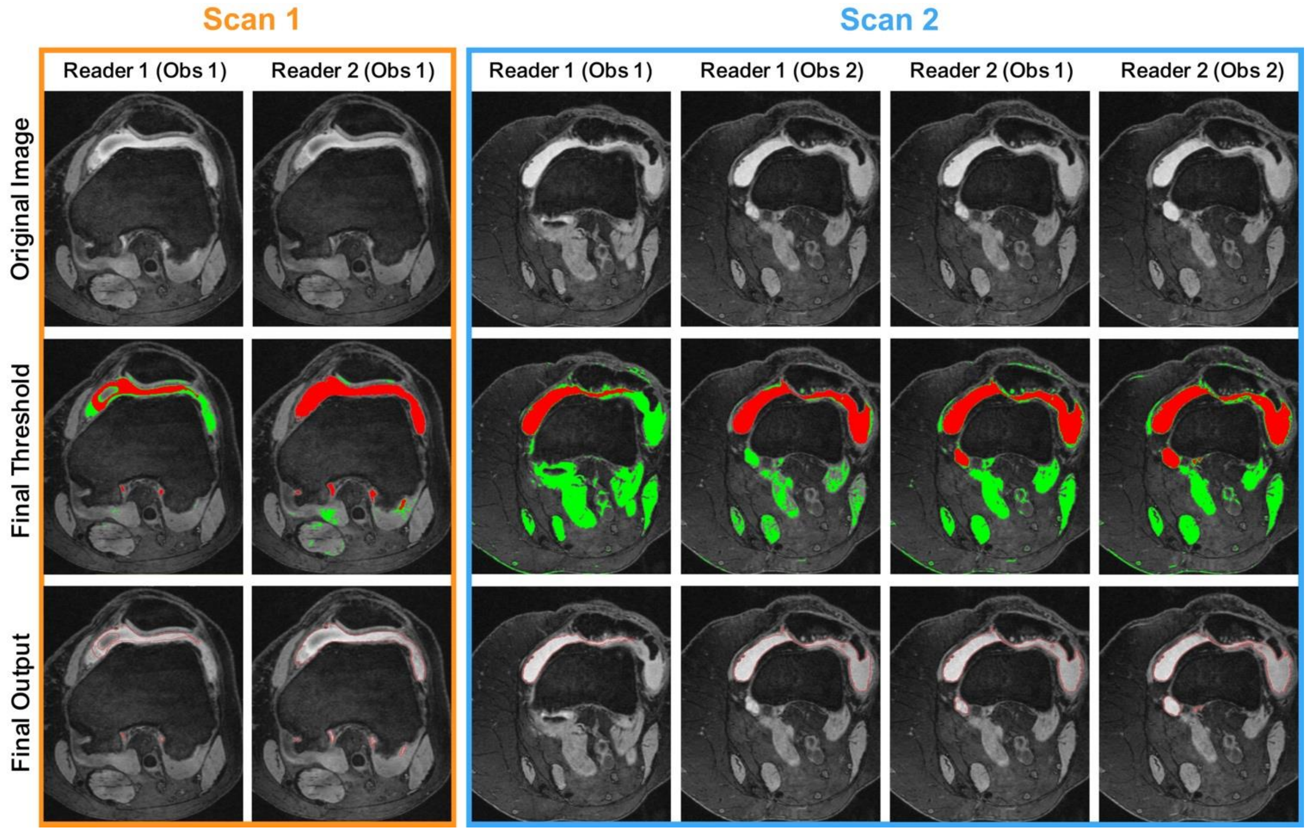

Figure S3. Two illustrative examples of variable threshold segmentation performance by non-expert readers**.** For some scans, effusion-synovitis (ES) will extend continuously from the medial to lateral recess across the patellofemoral joint, but the intensity of the signal will vary sub-regionally. The two scans presented in this figure were the most problematic regarding reader reliability but are readily addressable moving forward.

The threshold required to adequately measure ES found in the lateral regions of both scans would result in cartilage along the trochlea being segmented as ES if it was applied globally.

Key

Row 1: The single (unmarked) 3T axial MRI slice that was judged to contain the largest amount of ES and was segmented for ES by the reader for that scan/observation (Obs).

Row 2: Voxels with signal intensity exceeding the threshold set by the reader that are eligible for ES measurement are shown in green; voxels that have already been captured as ES are shown in red.

Row 3: The final segmented ES region(s) are shown with red outlines.

Columns 1-2 (outlined in orange) show one reading of the same scan made by two different readers.

Columns 3-6 (outlined in blue) show two readings (observations 1 and 2) of a different scan made by the same two readers; this scan appeared later in the data set.

Analysis

Column 1: The first reader set the final threshold too low and missed portions of fluid throughout the knee.

Column 2: The second (more experienced) reader provided a more accurate reading, fully capturing regions of fluid.

Column 3: The first reader set the threshold appropriately and captured the anterior-medial ES but missed a large lateral portion of ES (appearing in green).

Column 4: During the second observation, the first reader recognized the lateral fluid and captured it more fully, indicating that more exposure to scans may improve readers' ability to understand imaging features. Notice fluid in the posterior medial region was still missed.

Column 5: The second reader selected the same slice as the first reader had selected for their second observation (Column 4) and appropriately captured fluid throughout, including the posterior region.

Column 6: The second reader selected the maximum slice one slice earlier during their second observation (superior to the first), which had slightly less fluid anteriorly but a more noticeable increase in posterior fluid. This represents how the method's single-slice selection aspect introduced noise when making ES volume comparisons between readers/observations, most prominently affecting the computed subregional distributions.

A post-mortem analysis attributed the main error made on both scans by the first reader (who had the least relevant experience when joining the project) to the fact that the final training set did not include a case with subregional intensity variation of continuous ES occurring to this extent.
