# Supplementary figures and images for: Reliability and Validity of Single Axial Slice vs. Multiple Slice Quantitative Measurement of the Volume of Effusion-Synovitis on 3T Knee MRI in Knees with Osteoarthritis
Source: J Clin Med. 2023 Apr 4;12(7):2691. doi: 10.3390/jcm12072691 (PMC10095125; doi:10.3390/jcm12072691)

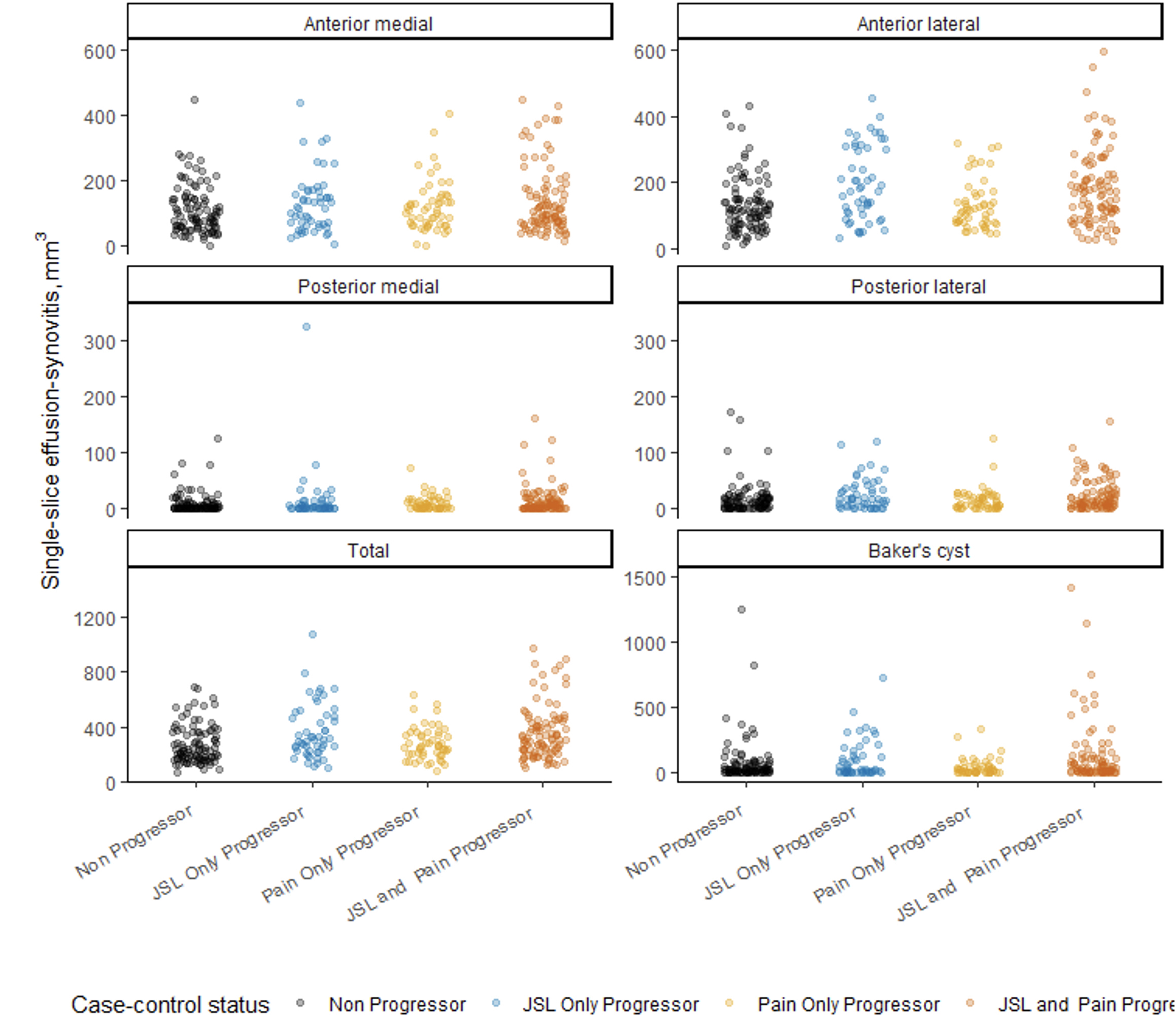

Supplement: Supplementary file 1 [file jcm-12-02691-s001.zip › Gilles - Figure S1. Distribution of single-slice volume by FNIH case status.jpg]

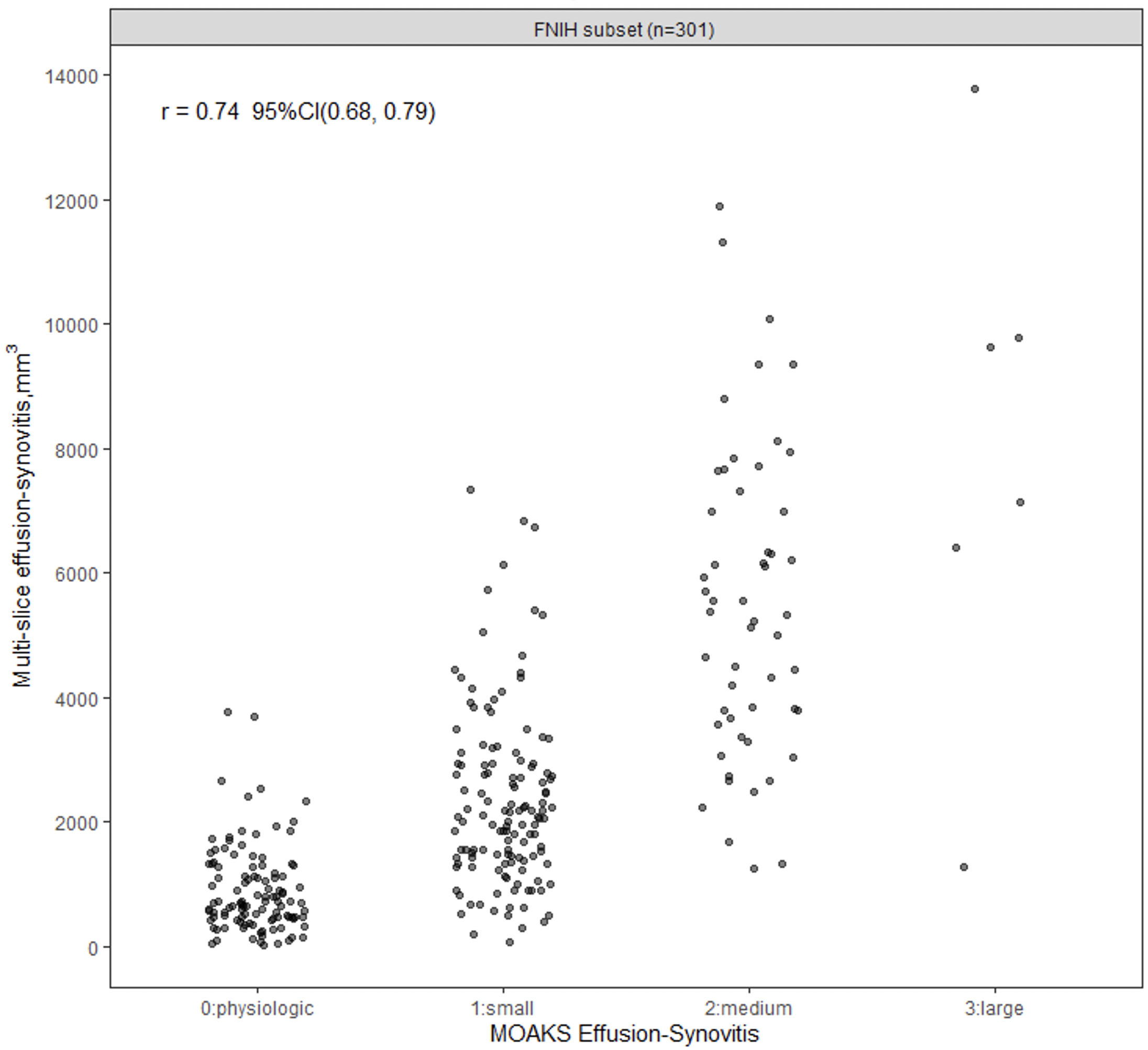

Supplement: Supplementary file 1 [file jcm-12-02691-s001.zip › Gilles - Figure S2. Multi-slice effusion-synovitis volume vs. MOAKS effusion-synovitis.jpg]
